# Novel insights into regulation of butyrophilin molecules: critical components of cancer immunosurveillance by γδ T cells

**DOI:** 10.1038/s41423-024-01138-w

**Published:** 2024-03-26

**Authors:** Dieter Kabelitz

**Affiliations:** Institute of Immunology, University of Kiel, Kiel, Germany

**Keywords:** Tumour immunology, Oncology

Members of the butyrophilin (BTN) family of transmembrane molecules play an important role in the activation of human γδ T cells. In recent years, it has been discovered that BTN3A1 and BTN2A1 are indispensable in this process, but other members like BTN3A2 and BTN3A3 are also required. So far, very little is known about the transcriptional and post-translational regulation of BTN expression. In a paper published in this issue, Wu and coworkers provide novel insights by demonstrating the importance of a specific transcription factor complex and the role of post-translational pyroglutamate modification for cell surface expression of BTN3 molecules [1]. These new results have important implications for our understanding of how human γδ T cells can recognize and kill tumor cells.

γδ T cells comprise a numerically small subset of CD3^+^ T cells in the blood but account for a major population of T cells in tissues including the gut. Two prominent features distinguish γδ T cells from the conventional CD4 and CD8 T cells expressing the αβ T-cell receptor (TCR): (i) γδ T cells do not require MHC/HLA molecules for TCR-dependent antigen recognition (i.e., they are not MHC/HLA-restricted); and (ii) γδ T cells do not recognize peptides presented by MHC/HLA molecules but rather a range of stress-inducible non-peptide ligands not seen by other immune cells [2]. The major subset of human peripheral blood γδ T cells expressing the Vγ9Vδ2 TCR (termed Vδ2 T cells in the following) recognizes phosphorylated intermediates of the mevalonate (eukaryotes) or non-mevalonate (prokaryotes) pathways of isoprenoid synthesis, termed phosphoantigens (pAg). The mevalonate pathway of cholesterol synthesis is frequently dysregulated upon cellular transformation, leading to increased pAg accumulation in cancer cells which renders them susceptible to recognition and killing by Vδ2 T cells [3]. Importantly, the endogenous production of pAg in tumor cells can be easily stimulated by aminobisphosphonates (like zoledronic acid, ZOL), drugs which are in clinical use for treatment of bone diseases and bone metastases [4]. ZOL pre-treatment thus sensitizes tumor cells to Vδ2 T-cell killing, and ZOL application in vivo or adoptive transfer of ZOL-expanded Vδ2 T cells has been used as immunotherapeutic approach in small scale clinical studies [5]. Despite the limited success of these early studies, γδ T cells have recently raised great interest for application in cancer immunotherapy [6, 7].

While the activation of Vδ2 T cells by pAg does not involve HLA molecules, there is an indispensable requirement for transmembrane molecules of the BTN family, specifically BTN2A1 and BTN3A1/BTN3A2/BTN3A3. Endogenous pAg bind to the cytoplasmatic B30.2 domain of BTN3A1 which subsequently interacts with BTN2A1 where pAg act as a glue [8]. In addition, γδ TCR triggering also involves the extracellular domains of BTN3A2/BTN3A3 [9]. Therefore, it is obvious that the recognition (and subsequent killing) of tumor cells by Vδ2 γδ T cells not only requires the (over)production of pAg by tumor cells (which can be further enhanced by ZOL) but also the adequate cell surface expression of the various BTN isoforms. So far, however, little is known about the transcriptional and posttranslational regulation of BTN surface expression. Insufficient expression of these molecules will reduce tumor susceptibility to Vδ2 T-cell killing (even if pAg are overproduced) and may contribute to tumor escape of Vδ2 immunosurveillance.

**Fig. 1:**
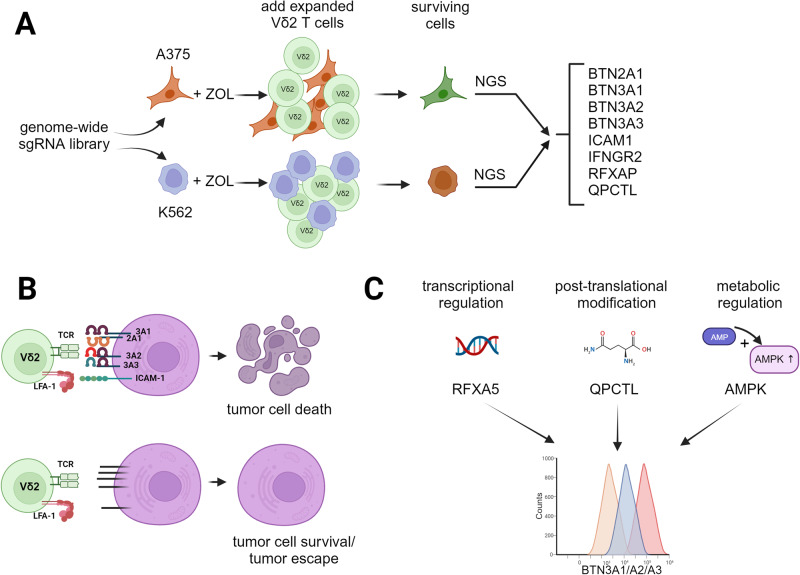
Genome-wide CRISPR/Cas9 screening revealed key components of tumor cell recognition by Vδ2 T cells. **A** Schematic workflow used by Wu et al. [1] to identify key molecules regulating tumor cell killing by in vitro expanded Vδ2 T cells using two independent tumor cell lines (melanoma A375, erythroleukemia K562). Top ranked hits identified in both screenings are listed on the right. **B** Individual knock-out of each of four BTN molecules (2A1, 3A1, 3A2, 3A3) or ICAM-1 drastically reduced tumor cell killing by Vδ2 T cells. **C** Wu et al. further identified transcriptional regulation of BTN molecules by the winged-helix transcription factor RFX5 and posttranslational pyroglutamate modification by QPCTL [1]. Recent additional data from Mamedov et al. indicate that BTN expression is also metabolically regulated by AMP-activated protein kinase (AMPK) [10]. Figure was created with BioRender.com

In an elegant study published in this issue, Wu et al. addressed this question by performing a genome-wide CRISPR screening in two cancer cell lines, A375/Cas9 melanoma and K562/Cas9 erythroleukemia. Cancer cells were infected with lentiviral sgRNAs, pretreated with ZOL to increase their susceptibility and then exposed to in vitro expanded Vδ2 T cells. Surviving tumor cells were exposed to a second round of killing by Vδ2 T cells. γδ T-cell resistant tumor cells and controls were then subjected to NGS sequencing. The two screenings revealed an overlapping set of genes which were differentially expressed in susceptible and resistant cancer cells, including both cell surface molecules (notably all above mentioned BTN isoforms and ICAM-1) and transcription factors/enzymes (Fig. 1A). Next, they used knockout cells of each individual molecule to delineate their relevance for Vδ2 T-cell recognition. Quite remarkably, the deletion of any of the four BTN isoforms BTN2A1, 3A1, 3A2 or 3A3 conferred protection to Vδ2 T-cell mediated killing (Fig. 1B). In line with the known significance of the ICAM-1/LFA-1 cell adhesion pathway for the interaction of Vδ2 T cells with tumor target cells, it was not surprising that deletion of ICAM-1 in tumor cells also led to tumor evasion (Fig. 1B).

Furthermore, the genome-wide CRISPR screening revealed novel molecular regulators of BTN3 expression, specifically the winged-helix transcription factor regulatory factor X-5 (RFX5) and the glutaminyl-peptide cyclotransferase-like (QPCTL) protein. By ChIP-seq they found that RFX5 binds to the promoter region of BTN and RFX5 deletion mutants of K562 expressed significantly less BTN3A1/A2/A3 and were much less susceptible to lysis by Vδ2 T cells [1]. Moreover, the CRISPR screening also revealed an important and hitherto unknown role of post-translational modification of BTN proteins. Wu et al. found that the N-terminal glutamine in BTN proteins is subject to pyroglutamate modification by QPCTL, and QPCTL-knockout cells expressed less BTN3A on the cell surface while the total amount of BTN3A protein was preserved. Again, the defective pyroglutamate modification of BTN proteins was associated with reduced lysis by Vδ2 T cells [1]. Interestingly, the expression of BTN proteins is also regulated at the metabolic level. In a recently published paper which was also based on genome-wide CRISPR screens to identify cancer cell pathways relevant for γδ T-cell detection, Mamedov et al. discovered a role of metabolic pathways, specifically ATP-producing processes, in regulation of BTN3A. The induction was found to depend on AMP-activated protein kinase (AMPK) [10]. Therefore, these exciting new results shed new light on the multifaceted molecular regulation of BTN3A cell surface expression (Fig. 1C).

Taken together, it is clear by now that the sensitivity of tumor cells to TCR-dependent recognition and killing by Vδ2 T cells is controlled by at least two parameters, i.e. intracellular accumulation of pAg and regulated surface expression of BTN molecules. While tumor cells can be sensitized by Zol and related aminobisphosphonates through accumulation of pAg, the new results discussed here will also spur the interest to design strategies for stabilization and/or increased cell surface expression of BTN molecules. This might be an important step towards improving the efficacy of γδ T-cell based immunotherapies [6, 7].
